# Patient, sufferer, victim, casualty or person with cervical myelopathy: let us decide our identifier

**DOI:** 10.1136/ihj-2019-000023

**Published:** 2020-06-25

**Authors:** Timothy F Boerger, Benjamin M Davies, Iwan Sadler, Ellen Sarewitz, Mark R N Kotter

**Affiliations:** 1 Physical Therapy, Marquette University, Milwaukee, Wisconsin, USA; 2 Addenbrooke's Hospital, Cambridge, Cambridgeshire, UK; 3 Myelopathy.org, Cambridge, UK; 4 The Goffin Consultancy, Canterbury, UK; 5 Welcome Trust-Medical Research Council Cambridge Stem Cell Institute, University of Cambridge, Cambridge, UK

**Keywords:** attitudes, patient advocacy, patient preference

## Abstract

Among biomedical journals, person-first language is considered preferable to identity person-first language. However, not all populations of people with certain medical diagnoses, such as deafness, prefer person-first language. Moreover, adherence to person-first language is poor among the literature on some neurological diagnoses. Therefore, it is most appropriate to consult with people with a given diagnosis regarding their preferred identifier. Here, we describe a consensus process undertaken by the REsearch objectives and COmmon Data Elements for Degenerative Cervical Myelopathy (RECODE-DCM, recode-dcm.com) steering committee members with cervical myelopathy to determine our preferred identifier.

The choice of language is important for effective communication, especially in healthcare. However, language by itself, for example, to denominate a group, can impact health outcomes.^1^

‘Person-first’ or ‘person-centred’ language refers to communication that acknowledges the individual first, followed by a descriptor of the condition or disability. It contrasts with ‘identity-first’ and/or ‘disease-first’ language, which highlight the condition.

Person-first language is advocated by many leading healthcare organisations, particularly in the context of disabilities, such as the National Institute for Health and Care Excellence (UK).^2^ However, certain groups, such as the ‘deaf’ community, prefer identity-first terminology, as they consider deafness a description and not a disability.^3^

Consequently, as part of AOSpine RECODE-DCM (www.recode-dcm.com), an international, multistakeholder consensus project to improve research efficiency we sought to confirm an appropriate identifier. This was left at the discretion of the eight members with DCM (six female, average age 56 and length of symptoms 6 years). Terms were suggested and/or opposed via email correspondence (summarised in table 1). Discussion quickly focused on person-first language, with eventual unopposed consensus for ‘Person with Cervical Myelopathy’. The term degenerative was dropped, but cervical retained, in order to offer a more concise description with sufficient specificity. The term was agreed by the full steering committee and adopted for AOSpine RECODE-DCM.

**Table 1:** Terms suggested by committee members with cervical myelopathy (n=8)

Term	N times suggested
Person with cervical myelopathy	6
Individual with cervical myelopathy	2
Patient with cervical myelopathy	1
Cervical myelopathy patient	1
Terms spoken against	Rationale (if given)
Sufferer	
Victim	
Patient	Only temporarily applicable (hopefully) Not reflective of ‘living life’ Carries connotation of disempowerment

The readiness to adopt person-first language appears variable,^1^ particularly in the scientific literature.^3^ This is despite its inclusion in numerous scientific style guides, including the American Medical Association Manual of Style.^4^ For example, despite the fact that persons with multiple sclerosis have adopted person-first language, of the 50 most highly cited papers of the past 5 years from Web of Science (Clarivate Analytics, USA), only 44% used person-first language.

The reason for this is unclear. However, it was interesting to note that despite our prior agreements, professionals drafting articles for early objectives of AOSpine RECODE-DCM, inadvertently returned to referring to individuals by their diagnosis or disability rather than using person-first language.

This topic has received contrasting commentary over the last 20 years.^5^ However, in an era of increasing user engagement in research design, conduct and consumption, we encourage community consultation on terminology and its consistent adoption, not just in ‘patient’ conversation, but throughout practice and research.

This is now the case for AOSpine RECODE-DCM.
